# Diversity is our Strength

**DOI:** 10.1117/1.NPh.8.3.030101

**Published:** 2021-09-29

**Authors:** Anna Devor

## Abstract

Neurophotonics Editor in Chief Anna Devor reflects on need to cherish and cultivate diversity within the global neuroscience and neurophotonics community by creating inclusive environments to welcome young students in sharing the joy of science.

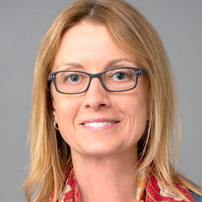



The first time I entered the Society for Neuroscience convention, I got a very strange feeling that all the ∼30,000 people at the convention center were members of my *karass*.^1^ It struck me suddenly without warning, and I was so surprised that I had to stop in my tracks – almost causing an accident in a busy entrance hall. The convention center where we were felt like a city, or maybe a continent, with people of every kind that have materialized at that point in space and time. Given my family’s mixed ethnic roots and religion, I failed to develop a strong cultural or geographic identity. But standing in that hall, there was no doubt in my mind that I was a citizen, I belonged. Throughout the years, I have retained this wonderful feeling about scientific meetings, big and small, with the *neuro* tracks at SPIE Photonics West and OSA Biophotonics rising to the top of my list in recent years.

For me, one of the biggest joys of living my life as a scientist is belonging to the global, international neuroscience and neurophotonics community. In this community, we differ in race, ethnicity, gender, sexual orientation, and culture. And this diversity is a secret ingredient for our success. Our unique experiences create distinct perspectives that generate dialog, pushing the community as a whole to think “outside the box,” and helping us sort out “rights” from “wrongs” in many emerging *neuro*-subjects including the field of *neuroethics*.

With all that said, we are living in times of divisive politics, persistent racial disparities, and a rise in anti-Asian racism fueled by the COVID-19 pandemic. These factors make it even more important to be aware and to use whatever platforms we have, no matter how small, to build inclusive environments where we all can share the joy of science. For the most vulnerable among us – our young students – a safe and welcoming environment is also key to preserving the pure curiosity and excitement that brings them into the world of science in the first place.

This summer, I taught an introductory neuroengineering class to a group of high school students. So, to avoid sounding abstract and generic, I would like to introduce to you three of these students by letting them describe their interest in neuroscience and neuroengineering in their own words.

**Figure:**
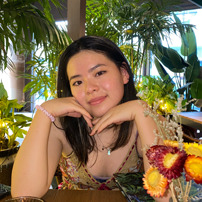
Chloe Chan

My name is Chloe Chan, I am a high school junior in Wallingford, Connecticut. I think that making memories and learning from the patterns we observe and remember is a survival mechanism that allows us to adopt a sense of order in an otherwise dynamic world. It is fascinating that so many diﬀerent parts of the brain contribute to the multiple aspects of a single memory. On the cellular level, if I recall a birthday party, a group of neurons allows me to remember where this party was. Another group of neurons would help me remember if we ate dinner or cake ﬁrst. However, when recalling a birthday party, I experience it more like I would experience watching my favorite movie. I see what has made it to the screen, but not all the work that went into creating the movie. The “behind the scenes” of memories is a complex and intricate process that I would like to understand, using modern neurophotonic technologies such as optical imaging of neuronal activity and optogenetics.

**Figure:**
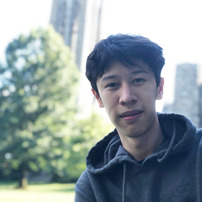
Nathan Chai

My name is Nathan Chai. I am a high school senior in Cincinnati, Ohio. I am most fascinated by the science behind how our brain functions. I would like to understand the mental/psychological processes behind one’s actions at a mechanistic biophysical level. I am in particular fascinated by the phenomenon of hallucinations, when a person perceives things that are not physically present. Hallucinations can be caused by drugs, such as LSD, or mental diseases, such as schizophrenia. However, heathy people can also experience imaginary sensory inputs. Originally stemming from my fascination for neurobiology, my interest in neuroengineering further grew after I read *The Power of Habit*. This book introduced me to the process of scientific research. I wish to engage in the area of neuroengineering and experience it ﬁrsthand, using brain imaging technologies.

**Figure:**
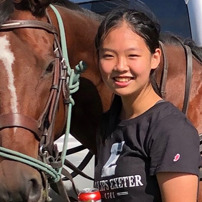
Sadie Shang

My name is Sadie Shang. I am a high school sophomore in Exeter, New Hampshire. I am especially interested in researching and learning more about Alzheimer’s and Parkinson’s disease because I love psychology and science and believe that a cure for these diseases will arrive in my lifetime. I would like to research possible treatments for these diseases, such as methods to produce more synaptic connections between neurons in the brain that would increase cognition and memory, diminishing the severity of neurodegeneration. I am also very interested in neurolinguistics and how damage to certain areas of the brain can aﬀect people in speciﬁc, diﬀerent ways. For example, people with Broca’s aphasia have difficulty speaking, but their comprehension is generally preserved. This is caused by damage to the anterior region of the brain and mostly aﬀects their sentences’ grammatical context. I would like to develop new neuroengineering methods, such as decoding of brain activity to generate words, to help these patients.
